# Reduced graphene oxide supported MXene based metal oxide ternary composite electrodes for non-enzymatic glucose sensor applications

**DOI:** 10.1038/s41598-022-24700-w

**Published:** 2022-11-29

**Authors:** Tamil Selvi Gopal, Khalid E. Alzahrani, Abdulaziz K. Assaifan, Hamad Albrithen, Abdullah Alodhayb, Muthumareeswaran Muthuramamoorthy, Saravanan Pandiaraj, Andrews Nirmala Grace

**Affiliations:** 1 Centre for Nanotechnology Research, Vellore Institute of Technology, Vellore, Tamil Nadu India; 2 Present Address: Biological and Environmental Sensing Research Unit, King Abdullah Institute for Nanotechnology, King Saud University, P.O. Box 2455, Riyadh, 11451 Saudi Arabia; 3 Department of Physics and Astronomy, College of Science, King Saud University, Riyadh, 11451 Saudi Arabia; 4 Department of Self-Development Skills, CFY Deanship, King Saud University, Riyadh, Saudi Arabia

**Keywords:** Nanoscale devices, Nanoscale materials

## Abstract

Diagnosis and monitoring of glucose level in human blood has become a prime necessity to avoid health risk and to cater this, a sensor’s performance with wide linearity range and high sensitivity is required. This work reports the use of ternary composite viz. MG–Cu_2_O (rGO supported MXene sheet with Cu_2_O) for non-enzymatic sensing of glucose. It has been prepared by co-precipitation method and characterized with X-ray powder diffraction, Ultraviolet–visible absorption spectroscopy (UV–Vis), Raman spectroscopy, Field emission scanning electron microscopy, High resolution transmission electron microscopy and Selected area diffraction. These analyses show a cubic structure with spherical shaped Cu_2_O grown on the MG sheet. Further, the electrocatalytic activity was carried out with MG–Cu_2_O sensing element by cyclic voltammetry and chronoamperometry technique and compared with M–Cu_2_O (MXene with Cu_2_O) composite without graphene oxide. Of these, MG–Cu_2_O composite was having the high defect density with lower crystalline size of Cu_2_O, which might enhance the conductivity thereby increasing the electrocatalytic activity towards the oxidation of glucose as compared to M–Cu_2_O. The prepared MG–Cu_2_O composite shows a sensitivity of 126.6 µAmM^−1^ cm^−2^ with a wide linear range of 0.01to 30 mM, good selectivity, good stability over 30 days and shows a low Relative Standard Deviation (RSD) of 1.7% value towards the sensing of glucose level in human serum. Thus, the aforementioned finding indicates that the prepared sensing electrode is a well suitable candidate for the sensing of glucose level for real time applications.

## Introduction

As per the World Health Organization (WHO) survey, a drastic increase in diabetics has made it as the 7th most deadliest and life-threatening disease in the world^1,2^, which is due to the insufficient amount of insulin in human body. In order to avoid this, periodic and continuous monitoring of glucose level (in human blood or tear or saliva or acetone level in breathing air) is necessary and is done by painless method such as non-invasive or closed loop technique^3,4^. Further economical and efficient sensors are required to be replaced with the currently available high-cost sensor in the market, which are enzymatic based leading to high cost, poor stability and reproducibility in the measurements^5^. An aforementioned issue with the enzymatic sensor is due to the denaturation of enzymes with temperature, pH and humidity ^6^. In order to resolve the pre-existing problems, huge effort is made by the researchers to develop enzyme free glucose sensors by direct oxidation of glucose molecule on the surface of the sensing electrode using metal or metal alloys as a sensing element (Pt, Pd, Au, Pt–Au, Ni–Cu, Pt–Pd and Ni–Cu)^7–9^. Even though it resolves the enzymatic sensor drawbacks, it still lacks in terms of production cost, selectivity and slow kinetic mechanism during glucose oxidation^10^. In order to overcome the above-mentioned issues, researchers are focusing on various new electrode materials especially metal oxides (MO) as a sensing element instead of metal-based electrodes. Various MOs such as CuO, Cu_2_O, Co_3_O_4_, NiO, and Fe_2_O_3_ have been investigated as alternative sensing elements^11–16^. Among the different metal oxides, copper-based metal oxide shows good catalytic activity towards glucose sensing for non-enzymatic sensor. But a lagging of sensing parameters such as sensitivity or linearity is observed and to avoid such lagging of sensing parameters, the active surface area or surface to volume ratio of the sensing elements has to be improved by fine tuning the size, shape, faces or with the use of composites with carbon-based materials and Cu/Cu_2_O heterostructures. Such reported investigations are listed in Table S1 and from the table, it’s clear that these kind of variations improve the sensitivity but not the linearity range of the sensor, which is also a prime requirement for diabetic patients.

Low conductive nature of single metal oxide may hinder the flow of electrons between the catalyst and electrode, which will further reduce the sensing performance of non-enzymatic glucose sensors. To cater this, highly conductive materials in combination with metal oxide as a binary or ternary or multi compound-based composites have been proposed ^17^. Various works on metal with MO (Pt/CuO, Co_3_O_4_@Pt@MnO_2_, Au/CuO, NiO–Au) ^18–22^, MO with MO (Co_3_O/NiO, ZnO/NiO, CuO/Cu_2_O, CuO/ZnO), ternary MO (Mn–Cu–Al), ^23–27^ carbon materials with metal oxide composites (CuO/rGO, Cu_2_O/rGO, NiO/rGO, NiCo@f–MWCNT, MWCNT/Fe_3_O_4_)^28–32^ have been studied as non-enzymatic glucose sensing elements. Among all these, reduced graphene oxide (rGO) based composites possess fast electron transfer ability during the oxidation of glucose due to its highly conductive nature, high surface area and good corrosion properties. In addition, composite of rGO with metal oxides improves the stability of the electrode as compared to other combinations. Zeng et al., prepared nickel oxide decorated reduced graphene oxide sheet for sensing of glucose molecules, which has shown a higher response as compared to bare NiO with a linear range of 0.005 to 4.5 mM. rGO sheets in the composite prevent the growth of clustered nanoparticles thereby enhancing the electron transfer ability between the catalyst and electrode^33^. Phetsang et al*.* prepared copper based rGO composites film for glucose sensing application. The prepared film showed a sensitivity of 172 µAmM^−1^ cm^−2^ with a linear range of 0.1 to 12.5 mM. Recently, Liu et al*.* prepared a three-dimensional metal oxide/metal/reduced graphene oxide complex as a sensing element. The prepared complex showed a higher response as compared to bare Cu_2_O with a linear range of 16.65 mM of glucose concentration^34^. Similar process was reported in various literatures in which different combination of rGO with metal oxides have been studied to improve the sensing performance of non-enzymatic glucose sensor^17,35–38^. Although these composites have improved the sensitivity as compared to bare metal oxides, it still needs a lot of improvement in the linear range with high sensitivity to make it commercially viable.

Recently, a new 2D material called MXene has been explored widely as an alternative to graphene and such materials are shown to be good for sensing applications typically in the linearity range. Among the different MXenes, titanium carbide (Ti_3_C_2_T_x_) has shown to be the best supporting material for electrochemical sensors due to its biocompatibility, high surface area, high porosity, high conductivity as well as the presence of a large number of functional groups on the MXene sheet^39–41^. These properties of Ti_3_C_2_T_x_ sheet helps to construct the best supporting material in the composites as compared to reduced graphene oxide for the preparation of non-enzymatic biosensors. Rakhi et al*.* prepared Au/Ti_3_C_2_T_x_ based composite for sensing glucose molecules with a wide linear range of 0.1 to 18 mM^42^. Chen et al*.* prepared Ce-metal organic framework with Ti_3_C_2_T_x_ composite for the sensing of L-Tryptophan and reported a wide linear range (0.2 to 139 µM) as compared to the carbon-based composites. As similarly, in our previous study, metal oxide (Cu_2_O) with Ti_3_C_2_T_x_ composites was shown to possess a broad linear range (0.01 to 30 mM) of glucose sensing with a good stability^43^. However, a number of works have been reported on the synthesis of composites with metal oxide, which suffer in terms of low sensitivity due to restacking or internal sheet aggregation, which in turn limits the access of target molecules during the electrochemical process. To prevent this, different strategies have been explored like introduction of foreign atoms or incorporating carbon-based materials in the MXene based composite structure^44–47^.

Based on these concerns, this work is focused on the preparation of Cu_2_O/rGO/Ti_3_C_2_T_x_ composite-based sensing elements by co-precipitation method. Further, the prepared material was characterized using XRD, Raman Spectroscopy, FE-SEM, HR-TEM and UV–Visible spectroscopy. The sensing element was fabricated as electrode probe and further employed to investigate the sensing of glucose using chronoamperometry (CA) technique.

## Experimental methods

### Chemicals and reagents

Graphite (325 mesh size), potassium permanganate (KMnO_4_), copper (II) acetate hydrate (Cu (CH_3_COO)_2_·H_2_O), fructose (C_6_H_12_O_6_), sucrose (C_12_H_22_O_11_), L-ascorbic acid (C_6_H_8_O_6_), sodium hydroxide (NaOH), sodium chloride (NaCl), potassium chloride (KCl), urea (CH_4_N_2_O), uric acid (C_5_H_4_N_4_O_3_) and D-glucose (C_6_H_12_O_6_) were purchased from Sigma Aldrich and the MAX phase was purchased from Forsman. Hydrofluoric acid (HF), sulfuric acid (H_2_SO_4_), hydrogen peroxide (H_2_O_2_), ethanol (C_2_H_5_OH) and dimethyl sulfoxide (DMSO) were purchased from SD Fine Chem Ltd. All the chemicals are of AR grade and utilized as it is without any further purification.

### Synthesis and fabrication of sensing electrode

#### Preparation of graphene oxide (GO)

The preparation of GO was carried out by modified Hummer’s method as per the previous reports^47,48^ with a slight modification. In brief, 2 g of graphite powder was added to 100 ml of H_2_SO_4_ and stirred for 30 min. The mixed graphitic solution was then transferred to ice-bath and then 12 g of KMnO_4_ was added slowly. After 2 h, the solution was removed from ice bath and stirred at room temperature (RT) for 2 h. After this, 200 ml of ice water was added gradually and then heated at 50 °C for 2 h. After heating, the solution was kept to cool down to RT and then 600 ml of DI water was added slowly. Finally, 10 ml of H_2_O_2_ was added to it and stirred overnight. The obtained solution was further washed with DI water until the supernatant becomes neutral. The solid was transferred to petri dish and dried at.

#### Preparation of MXene and MG composites

**Figure 1:**
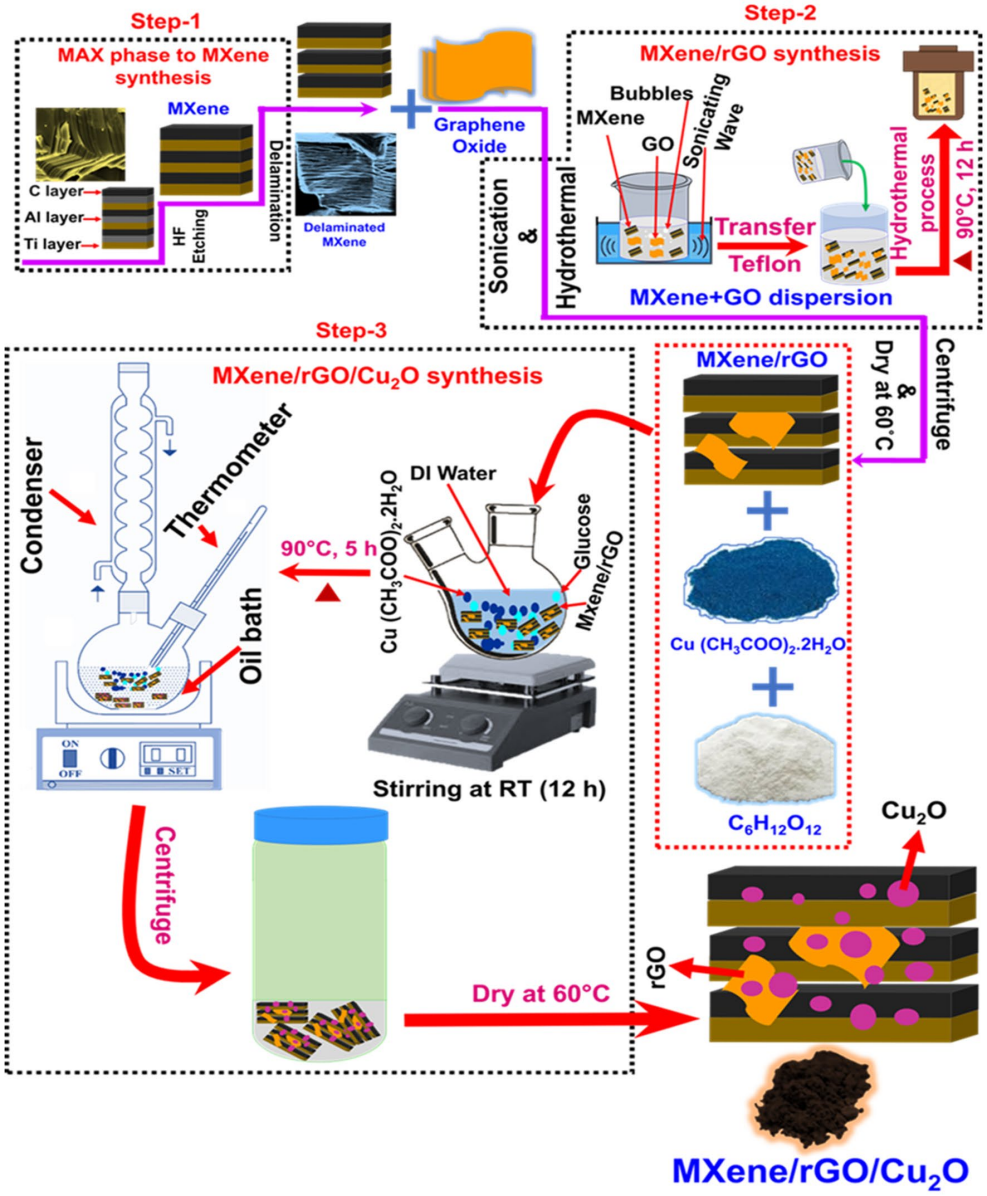
A schematic showing the synthesis process of MG–Cu_2_O composite.

The conversion of MAX phase to MXene was carried out as similar to our earlier report^43^ and the preparation of MG composite is given in Fig. 1 (step 1 and 2). The formed MXene was used for the preparation of MG composites by hydrothermal method. Initially, 100 mg of MXene was added to 90 ml of DI water and then sonicated for 30 min labeled as solution A. Similarly, 100 mg of GO was added into 90 ml of DI water and then sonicated for 30 min and named as solution B. The prepared solution B was added into solution A and sonicated for 1 h. After this, the mixed solution was transferred to 250 ml Teflon lined stainless steel autoclave and heated at 90 °C for 12 h. After that, the solution was cooled down to RT, the obtained solution is further centrifuged and washed with DI water several times. Finally, the formed rGO–MXene solid was dried at 60 °C and named as MG-100. Likewise, different weight ratio of graphene oxide in solution B was varied with a fixed amount of MXene (100 mg) for the preparation of MG composite and named as MG-0, MG-25, MG-50 and MG-75 respectively.

#### Preparation of Cu_2_O-rGO-MXene composite

The synthesis process of Cu_2_O–rGO–MXene composite is given in Fig. 1 (step 3). To 100 mg of the prepared MG-50 composites, 1 g of copper (II) acetate hydrate and 1.8 g of D-Glucose was added to 100 ml of DI water and sonicated for 1 h. The mixed complex solution was further stirred at RT for 12 h. After this, the solution was transferred to an oil bath and heated at 90 °C for 5 h. The obtained product was further washed with DI water and ethanol for 5 times and then dried at 60 °C overnight and labeled as MG–Cu_2_O. Bare MXene–Cu_2_O composite was prepared as similar to the earlier report for comparison and labeled as M–Cu_2_O ^43^.

### Characterization and electrochemical measurements of sensing materials

The formation of MG composite and crystallinity of Cu_2_O–rGO–MXene composite were confirmed with XRD (D8 Advance from Bruker, Cu Kα radiation) and Raman spectroscopy (512 nm laser—HORIBA). UV–Visible absorption spectrum was carried out with JASCO (V-670 PC) to examine the optical behavior of the prepared samples. The morphology and particle distribution of Cu_2_O nanoparticles on the rGO and MXene sheet in the composite was analyzed with FE-SEM (Thermo Fisher FEI Quanta 250 FEG) and HR-TEM (FEI-Tecnai G2 20 Twin). All the electrochemical measurements were carried out with CHI-600C workstation.

### Preparation of sensing electrode

Cu_2_O–rGO–MXene composite electrode was fabricated by drop casting method. Initially, the glassy carbon electrode was cleaned with Al_2_O_3_ powder and then washed with ethanol, acetone followed by DI water and the washed electrode was further dried under N_2_ gas. For the deposition of the prepared composite, a mixture of 1 mg MG–Cu_2_O, 0.045 ml of DI water and 0.005 ml of 5% nafion solution was sonicated for 30 min and then 5 µl of mixed solution was dropped on the dried electrode. Further the dropped electrode was left overnight at room temperature for drying. In the same way, MG composite and M–Cu_2_O composites-based electrodes were prepared and then further used for studying the sensing performance.

## Results and discussion

### Structural analysis of bare and composites

**Figure 2:**
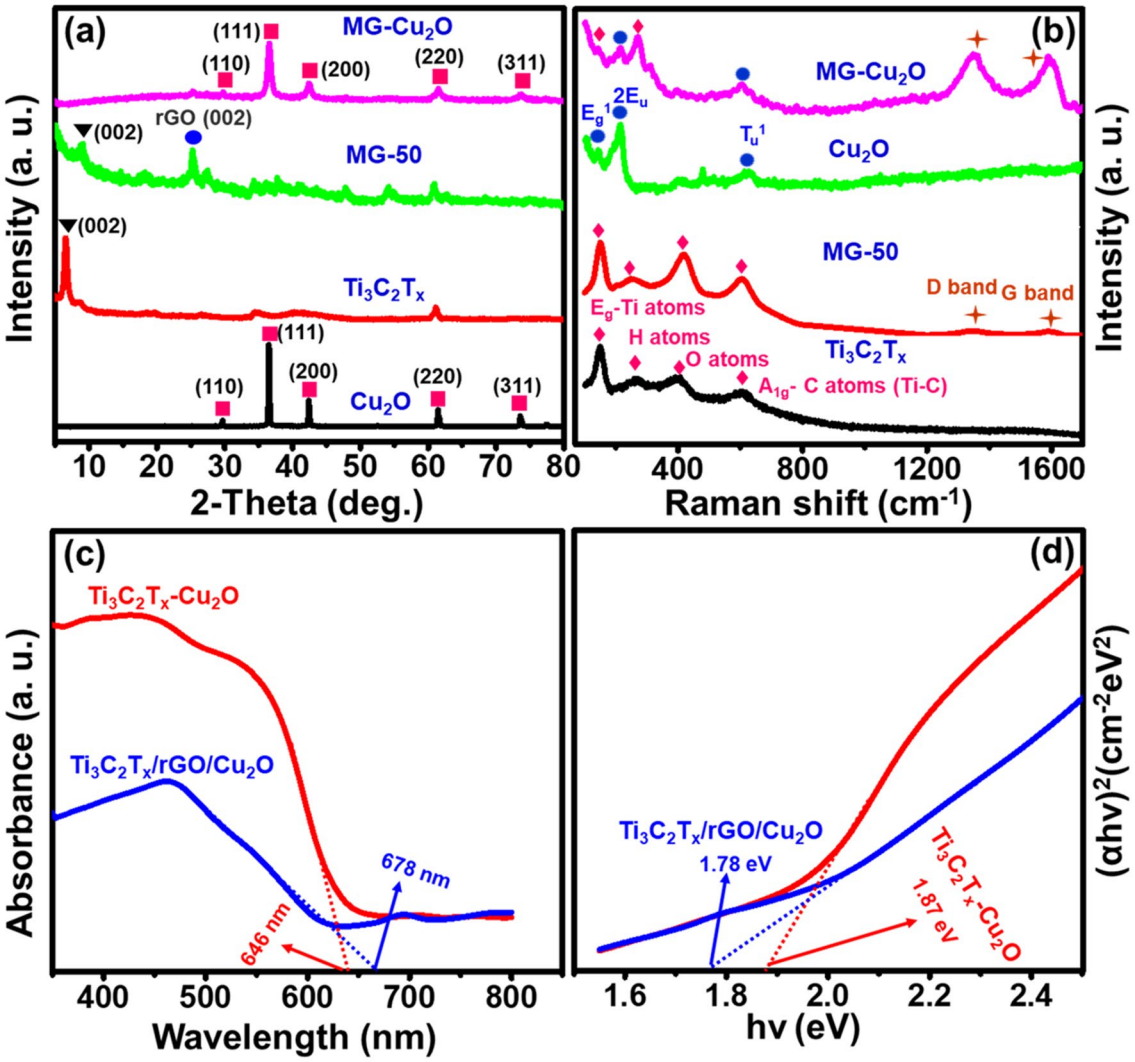
(**a**) X-ray diffraction patterns of synthesized Cu_2_O, Ti_3_C_2_T_x_, MG (50% of GO) and MG–Cu_2_O composite (**b**) Raman spectra for Cu_2_O, MXene, and their composites (**c**) Optical absorption spectra of M–Cu_2_O, MG–Cu_2_O and (**d**) Optical band gap for M–Cu_2_O, MG-Cu_2_O.

The conversion of MAX phase to MXene, MG and MG–Cu_2_O composite formation process is given in Fig. 1. To confirm the formation of the material, structural and phase analysis were carried out with XRD and Raman spectroscopy. For the bare MXene (MG-0), the disappearance of 38.92° peak (Al peak) as well as a shift of (002) peak from 9.54° to ~ 6.4° confirms the formation of MXene from MAX phase as given in Figs. 2a, S1 respectively^49^. The XRD of the prepared MG composite at different loadings of graphene oxide (25 to 100% of GO) is given in Fig. S1. The broadening of (002) peak as well as a shift from ~ 6.4° to ~ 8.8° and appearance of rGO peak at 25.35° confirms the formation of reduced graphene oxide with MXene composite^50^. As the loading amount of graphene oxide increases from 25 to 100%, an increment in the intensity of reduced graphene oxide peak as well as the clamp down of MXene peaks are observed. The corresponding d-spacing values of MXene sheet in the MG composites are 13.8 Å (MG-0), 10.14 Å (MG-25), 9.98 Å (MG-50), 10.37 Å (MG-75) and 10.1 Å (MG-100) respectively.

For the MG–Cu_2_O composite, XRD peaks are observed at 2θ values of 29.5°, 36.4°, 42.3°, 61.3° and 73.5° respectively matching with the JCPDS (Joint Committee on Powder Diffraction Standards) card number 05–0667 with cubic Cu_2_O^43^ and the corresponding faces are (110), (111), (200), (220) and (311). The obtained XRD peak of Cu_2_O in the composite is similar to bare Cu_2_O but the broadening of peak is observed, which indicates the reduction of crystalline size in the composite (14.33 nm) as compared to bare (35.69 nm).

To understand the functional groups and confirmation of the reduction of graphene oxide to reduced graphene oxide, FTIR analysis was done and given in Fig. S4. A broad frequency range observed around 3200–3650 cm^−1^ is related to the –OH stretching vibration. For Ti_3_C_2_T_x_, graphene oxide and Cu_2_O, a peak at 2985 cm^–1^ (C–H stretching of methylene), 2894 cm^−1^ (C–H stretching of methyl functional group), 1724 cm^−1^ (C=O stretching), 1620 cm^−1^ (C=C), 1045 cm^−1^ (stretching vibration of epoxy bond (C–O bond)), 947 cm^−1^ (C–F) and 620 or 626 cm^−1^ (Cu–O stretching vibration of Cu_2_O) was observed. For the composites (rGO–Cu_2_O, MG–Cu_2_O), the disappearance of C–O peak confirms the reduction of graphene oxide to reduced graphene oxide and highlighted in Fig. S4^49^. The conversion of GO to rGO occurs due to the presence of glucose in the reaction system, which acts as a reducing agent for both GO as well as for Cu_2_O during its synthesis^49,72^. In order to find the defect density of carbon material in the composites and confirmation of un-oxidized MXene in the composites, the material was studied with Raman spectroscopy (Figs. 1b, S2a and S2b). From Figs. 2b, S2a, peaks at 158, 288, 417 and 608 cm^−1^ are related to the in-plane vibration of Ti–C bond (E_g_ mode), surface terminal groups (–OH) and the out-plane vibration of Ti–C bond (A_1g_ mode), respectively^51^. Along with these, two additional peaks are also observed for MG composites and are related to the D-band (1350 cm^−1^) and G- band (1580 cm^−1^) of rGO as given in Fig. S2b^52,53^. A variation in the I_D_/I_G_ ratio is observed with an increment in the loading amount of GO, which is in the order of MG-50 (1.055) > MG-100 (1.038) > MG-75 (1.032) > MG-25 (0.827). A higher disorder intensity is observed for MG-50 composite due to the strong interaction or bonding between reduced graphene oxide and MXene sheet as compared to other composites^49,54,55^.

For MG–Cu_2_O, the combined peaks of Eg mode (Ti–C) of MXene, D-band and G-band of reduced graphene oxide and Cu–O vibrations (214 cm^−1^ (2Eu) and 628 cm^−1^ (Tu1)) are observed. It is worth to notice that there is absence of TiO_2_ (142 cm^−1^) peak, which signifies that MXene sheet in composites are not oxidized^43^. Further, the optical properties of bare and composites were studied with UV–Vis spectroscopy as given in Figs. 2c, S3a,c. From Fig. S3a, a strong adsorption band is noticed in the UV region of MXene and MG-50 composites, which is in agreement with the previous reported literatures^56^. It is related to the –OH functional group on the sheets, which leads to the bandgap formation of MXene (1.2 eV) and MG-50 composites (1.28 eV)^56,57^. From Figs. 2c, S3c, a red shift of the adsorption edge is observed for MG–Cu_2_O composite (~ 678 nm) as compared to the Cu_2_O (~ 635 nm) and M–Cu_2_O (~ 646 nm) composite.

The corresponding bandgap of material is obtained from the Tauc plot and is given in Figs. 2d, S3d. The bandgap values are 1.78 eV (MG–Cu_2_O), 1.87 eV (M–Cu_2_O) and 1.9 eV (Cu_2_O) respectively. The above findings suggest the formation of active heterojunction between the MG and Cu_2_O interface as well as a strong electron conduction of graphene in the MG–Cu_2_O composite^58^. As per the structural analysis through XRD, Raman and UV–Visible spectroscopy, a low d-spacing and high defect density of MG-50 composite lead to high conductivity as compared to MG-0, MG-25, MG-75 and MG-100 respectively. A low value in the crystallite size and a red shift in the adsorption edge of Cu_2_O in the composite may lead to more active sites as compared to bare Cu_2_O.

### Morphological study of bare materials and composites

**Figure 3:**
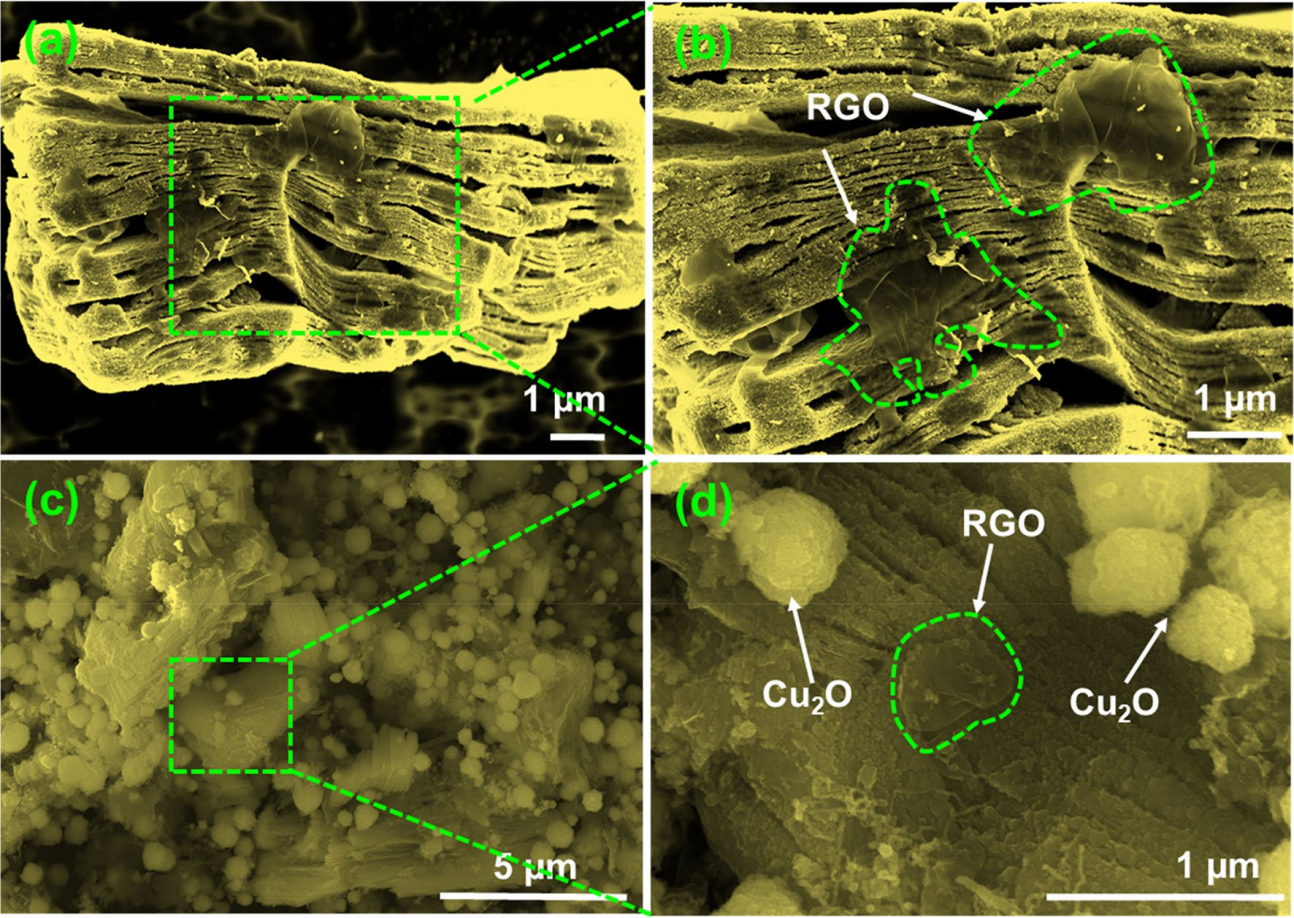
FE-SEM images at different magnifications (**a**, **b**) MG-50 and (**c**, **d**) MG-Cu_2_O composite.

Morphological study of MAX phase, MXene, MG-50 and MG–Cu_2_O composites were done with FE-SEM and given in Figs. S5 and 3. After HF etching of the MAX phases (Fig. S5a,b), a spacing between the layers are observed as similar to the accordion like structure and is given in Fig. S5c,d. This formation is one more evidence for MXene conversion as supported with the result of XRD (a shift of (002) peak from 9.54° to ~ 6.4° and disappearance of 38.92° peak). After sonication and hydrothermal treatment of MXene and graphene oxide sheets, the formed reduced graphene oxide sheets are crevice and cover the MXene sheets ^44^ (highlighted in green—Fig. 3a,b). It reveals that the two materials are well attached leading to the formation of MG composites^59^.

**Figure 4:**
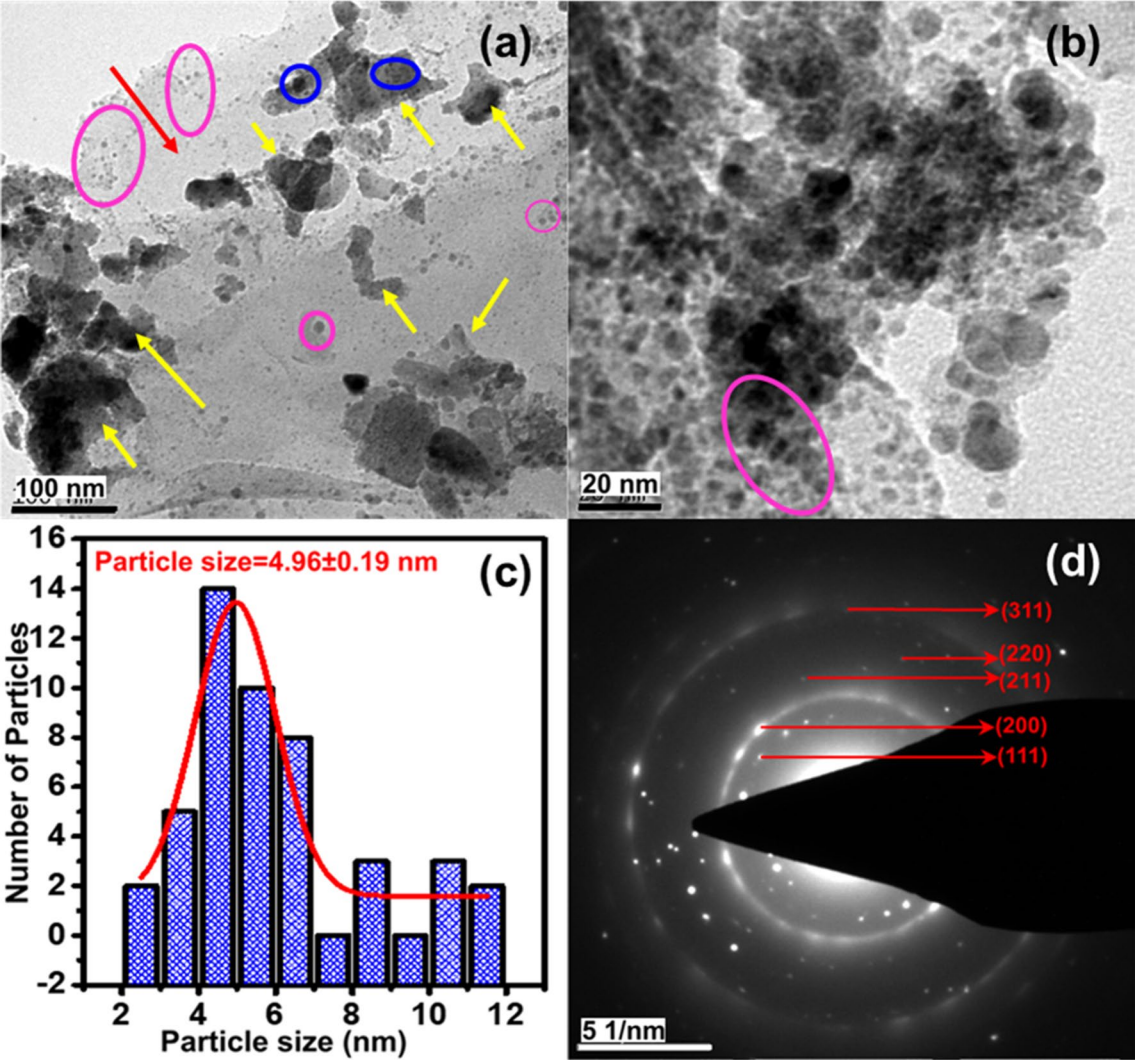
(**a**, **b**) HR-TEM images of MG-Cu_2_O composite with different magnification (Pink and blue circle shows the Cu_2_O on the edges of MXene and rGO sheet, the yellow arrow shows the rGO sheets in the composites and red arrow indicates the MXene sheet) (**c**) size distribution of Cu_2_O nanoparticles on MXene sheet (**d**) selected area electron diffraction (SAED) pattern of MG-Cu_2_O composite.

For the MG–Cu_2_O composite (Fig. 3c,d), a non-uniform distribution of aggregated Cu_2_O nanoparticles are ornamented on the surfaces of MG-50 composite. These particles are spherical in shape with the size of 500–700 nm. To confirm the role of rGO sheet in the morphological changes, a MG–Cu_2_O composite is prepared without rGO sheet (M–Cu2O) and analyzed with FE-SEM. In the absence of reduced graphene oxide sheet, an octahedron shape of Cu_2_O (size of 1.5 µm) is formed and highlighted in red circle in Fig. S6a. In contrast, a spherical shape (Figs. 3d, S6b) of Cu_2_O (size of 500–700 nm) is formed for MG–Cu_2_O composite. In the presence of rGO sheet, the preclusion of restacking the MXene sheets maintain the larger surface area of sheets as well as the ample amount of functional groups on the surface and edges of sheet may hinder the nanoparticle agglomeration leading to the formation of spherical shape of Cu_2_O in the MG–Cu_2_O composite as given in Fig. S6b (marked in red circle). In order to understand the distribution of Cu_2_O nanoparticles (MG–Cu_2_O), HR-TEM was done and given in Fig. 4a,b. A larger number of nanoparticles are embedded on the surface as well as edges of sheets as observed from Fig. 4b. The average sizes of the distributed nanoparticles are ~ 4.96 ± 1.9 nm and the respective histogram plot is given in Fig. 4c**.** Further, the nature of distributed nanoparticles is studied with the help of SAED pattern for MG–Cu_2_O composite (Fig. 4d). It shows five different rings along with different spots indexed with (111), (200), (211), (220) and (311), which are well matching the XRD results.

### Electrochemical properties of bare and composite electrodes

In order to find out the highest electron transfer rate capability of GO loaded MG composites (MXene, MG-25, MG-50, MG-75 and MG-100), cyclic voltammetry (CV) and electrochemical impedance spectroscopy (EIS) techniques were investigated in 0.1 M KCl with 5 mM of [Fe(CN)_6_]^4−/3−^ electrolyte (Fig. S7a–d). As observed from the figure, both anodic and cathodic peaks are observed in cyclic voltammetry profile of all the materials with the potential difference (ΔEvp) of 395 mV (MXene), 275 mV (MG-25), 179 mV (MG-50), 233 mV (MG-75) and 250 mV (MG-100) respectively. Of the various composites, 50 wt% of graphene oxide loaded MG composite (MG-50) shows a lower ΔEvp as compared to other materials, which indicates the high electron transfer rate as compared to others^60^. An increment in graphene oxide loading may reduce the active electrochemical surface area and hinder the flow of electrons, which is further confirmed with the charge transfer resistance value (Rct) between the modified electrode and electrolyte for these materials.

For obtaining this, an electrochemical impedance spectral analysis was carried out in the same medium and given in Fig. S7b,d. For all the wt% of graphene oxide loaded MG composites, the graph shows a semicircle along with straight-line in the high frequency region. The diameter of the semicircle is equivalent to the charge transfer resistance value^61^. The obtained values for Rct are 80 kΩ (MXene), 110 kΩ (MG-25), 0.25 kΩ (MG-50), 1.46 kΩ (MG-75) and 20 kΩ (MG-100) respectively. A low charge transfer value for MG-50 composite indicates the high electron transfer rate capability as compared to the other loading, which might be due to its low d-spacing and highest defect density of the prepared material MG-50.

**Figure 5:**
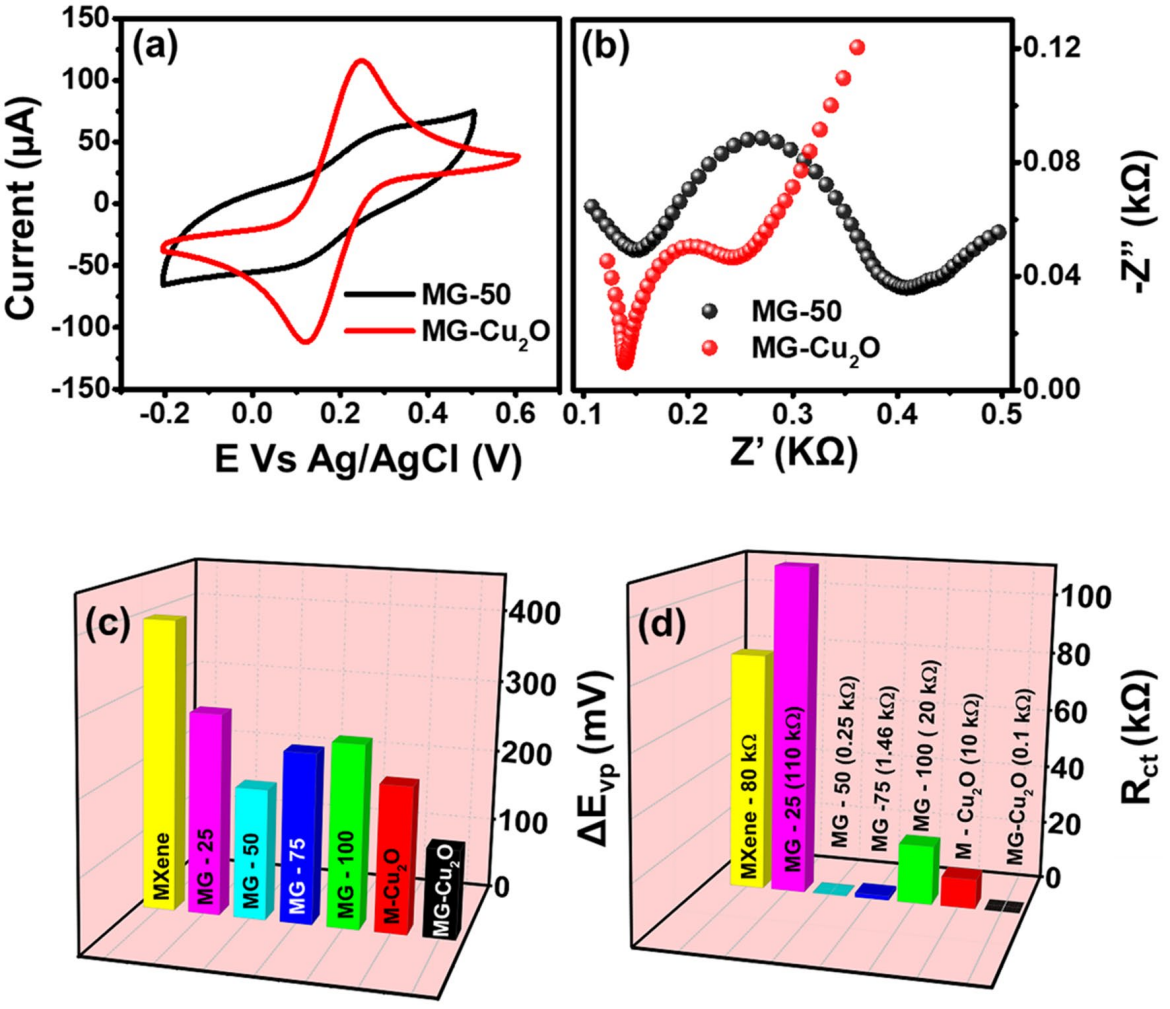
(**a**) Cyclic voltammetry comparison of MG-50 and MG-Cu_2_O composites in 5 mM Fe (CN)^3-/4-^ along with 0.1 M KCl (**b**) Nyquist plot of MG-50 and MG-Cu_2_O composites with the frequency range of 1 MHz to 0.1 Hz (**c**) 3D bar graph of potential difference between redox peak (ΔE_vp_) for different materials and (**d**) 3D bar graph of charge transfer resistance (R_ct_) for different materials.

**Figure 6:**
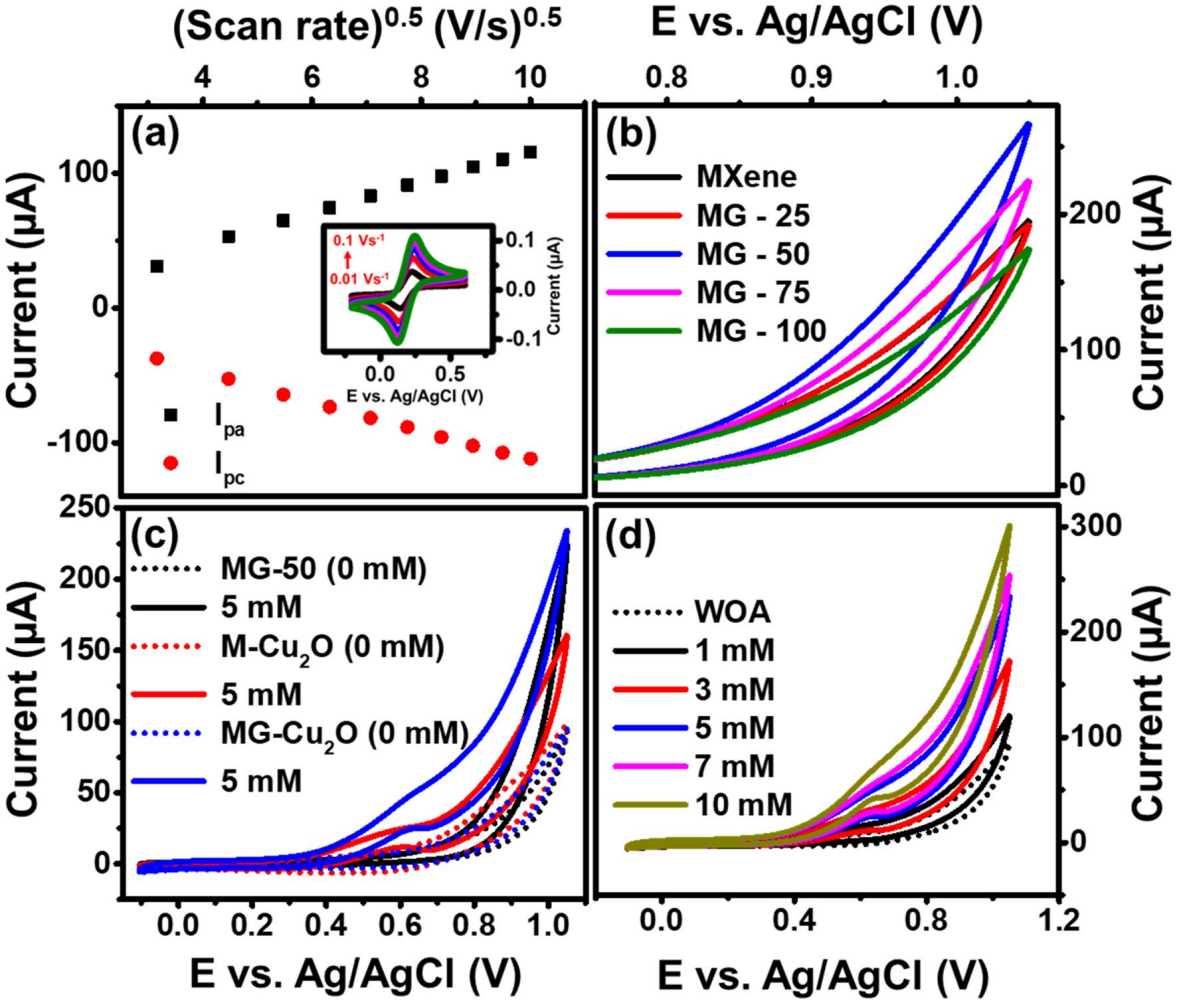
(**a**) Peak current vs. square root of scan rate for MG-Cu_2_O composite and the inset—cyclic voltammetry (0.1 M of KCl + 5 mM of Fe (CN)^3−/4−^solution) at different scan (**b**) CV response of MG composite with varying the weight ratio of MXene and Graphene oxide in the presence of 5 mM glucose (in 0.1 M NaOH) (**c**) CV curve of MG-50, M-Cu_2_O and MG-Cu_2_O composite at 0 and 5 mM glucose (in 0.1 M of NaOH) and (**d**) CV of MG-Cu_2_O composite at different concentrations of glucose in 0.1 M NaOH.

Further, MG-50 composite has been chosen for the preparation of ternary composites (MG–Cu_2_O composites). A cyclic voltammetry and electrochemical impedance spectra analysis of M–Cu_2_O (Fig. S7e,f), MG-50 and MG–Cu_2_O composite is given in Fig. 5a,b. The addition of Cu_2_O catalyst to MG-50 enhances its anodic and cathodic current as compared to MG-50 alone. A comparative plot of the voltage difference between redox peak (ΔE_vp_) and charge transfer resistance (R_ct_) for the different MG composites, M–Cu_2_O and MG–Cu_2_O are given in Fig. 5c,d. From this plot, a low ΔE_vp_ (121 mV) and R_ct_ (0.1 kΩ) value was observed for MG–Cu_2_O, which might be due to its interconnected structure of Cu_2_O with rGO and MXene sheets as supported with FE-SEM image (Fig. 3c,d). In order to understand the charge transfer mechanism, the diffusion studies was further performed for MG–Cu_2_O composite by tracing the peak current response with changing scan rates from 10 to 100 mV/s (Fig. 6a). A linear response is observed for peak currents with respect to the square root of scan rate, which indicates that the rate of the reaction is a diffusion-controlled process^62^.

### Electrocatalytic study of bare and composites electrode

**Figure 7:**
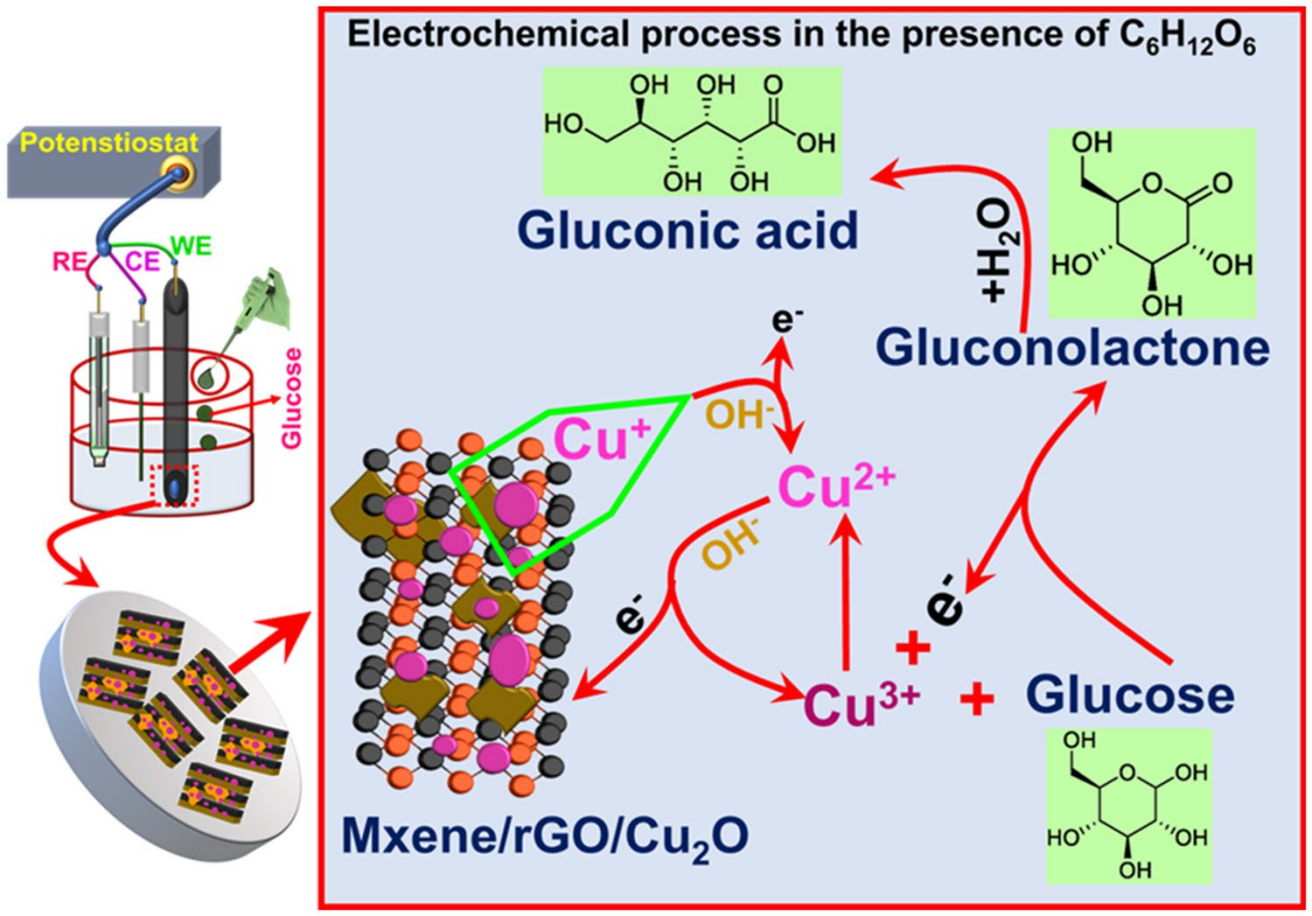
A schematic showing the glucose sensing mechanism of MG–Cu_2_O composite.

In order to verify the above-mentioned results, cyclic voltammetry study of MXene, MG-25, MG-50, MG-75 and MG-100 was carried out in 0.1 M NaOH in the presence of glucose and given in Fig. 6b. A higher current variation is observed for MG-50 composite as compared to others, which implies the faster electron transfer between the electrode and sensing element during the oxidation of glucose. Further, the role of rGO and Cu_2_O in MXene based composite is studied and given in Fig. 6c. No variation in the current is observed in the absence of glucose analyte for MG-50, M–Cu_2_O and MG–Cu_2_O respectively. With the addition of 5 mM glucose, instantaneous enhancement in the current was observed for all the materials, which signifies that all the prepared materials can act as a sensing element for glucose sensor. Among the composites, a huge enhancement was observed when bare M–Cu_2_O is replaced with MG–Cu_2_O (Fig. 6c). There is a presence of anodic peak in the potential range of 0.4 to 0.8 V, which occurs due to the oxidation of copper ion in the electrode during the sensing of glucose molecule as explained in Fig. 7. Briefly, in the presence of glucose, Cu^+^ ions are oxidized to Cu^2+^ with the release of electrons into the solutions and the formed Cu^2+^ ions are further oxidized to Cu^3+^ ions. These processes initiate the oxidation of glucose and simultaneously there is a reduction of Cu^3+^ to Cu^2+^ resulting in an enhancement of current. Further, the formed gluconolactone gets converted to gluconic acid at 0.6 V via the hydrolyzation process^63^. In the composite, rGO prevents the stacking of MXene sheet, which improves electron transfer rate with more active sites. This may enhance the peak and spike current of MG–Cu_2_O composite, indicating more Cu^3+^ ion formation for MG–Cu_2_O composite as compared to M–Cu_2_O. As the concentration of glucose increases, a spike and peak current of MG–Cu_2_O modified electrode increases. It shows that the prepared composite is electrochemically active and responding well during the oxidation of glucose. Therefore, the replacement of M–Cu_2_O to MG–Cu_2_O composite has enhanced the glucose oxidation current beneficiary for sensor applications.

To reinforce the above-mentioned result (CV analysis), a chronoamperometry (CA) analysis of M–Cu_2_O and MG–Cu_2_O composite was carried out in 0.1 M NaOH at different concentrations of glucose (Fig. S8a). As the concentration of glucose increases, a huge improvement in current for MG–Cu_2_O (Figs. 6d, S8a) was observed as compared to M–Cu_2_O. Therefore, results show that the replacement of M–Cu_2_O with MG–Cu_2_O increases the conductivity of the sensing element and improves the active sites for sensing analyte.

### Sensing performance of modified electrode

**Figure 8:**
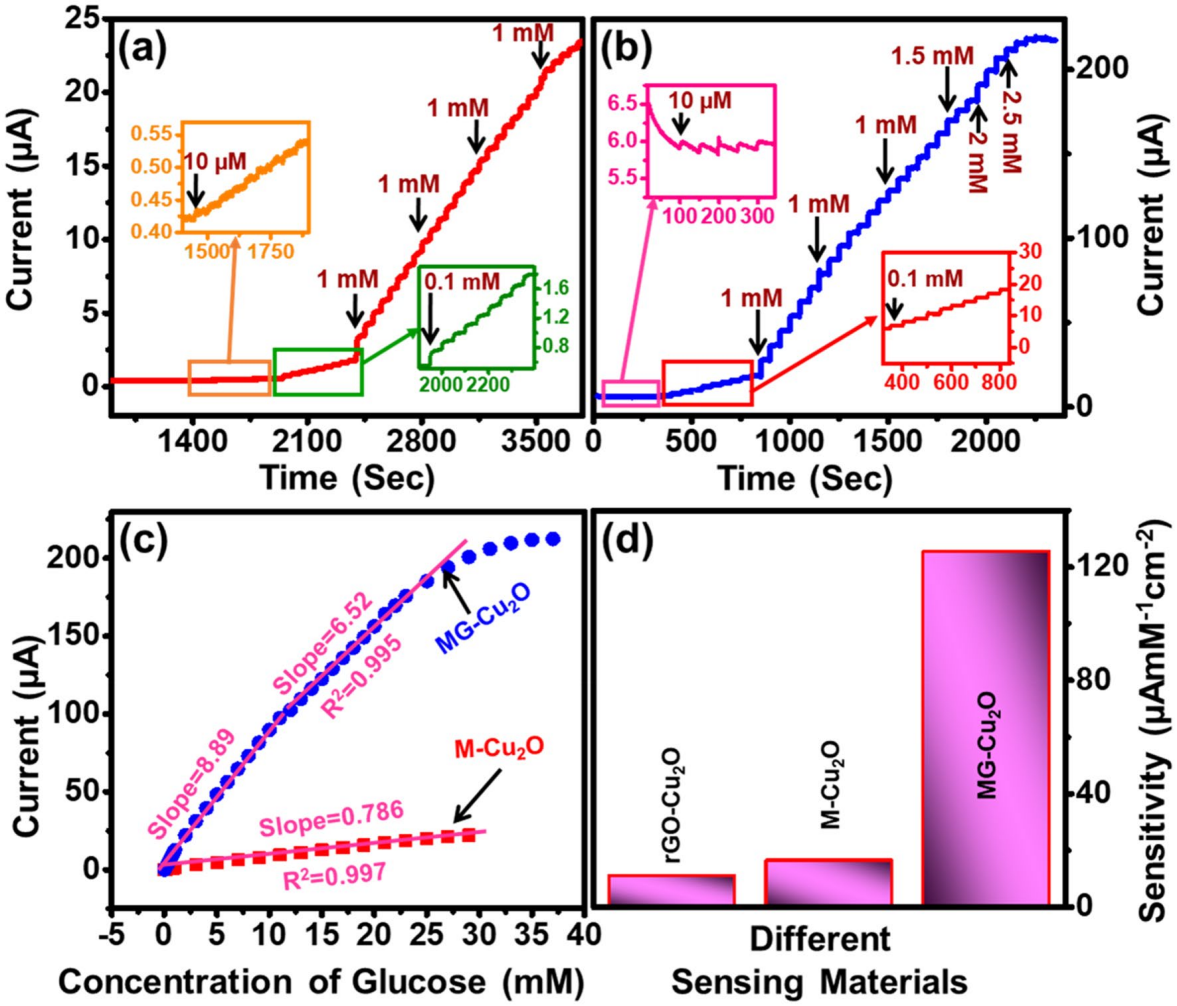
(**a**, **b**) Current versus time (Chronoamperometry) study of M–Cu_2_O and MG–Cu_2_O composite with the successive addition of glucose at 0.6 V versus Ag/AgCl (inset—zoomed at lower concentrations) (**c**) Comparative linear plot of MG–Cu_2_O and M–Cu_2_O composite (**d**) Sensitivity of prepared sensing materials.

In order to determine the suitable working potential of MG–Cu_2_O, CA study was further carried out with the addition of 0.1 mM glucose in 0.1 M NaOH at various bias voltages (0.5, 0.6 and 0.7 V). The corresponding current response is plotted with respect to concentration of glucose and given in Fig. S8b. An increment in the current is observed with an increase in the concentration of the glucose at all the biasing conditions. Among all the bias, a high change in the current was observed at 0.6 V and hence this potential was kept constant for further study. The sensitivity of MG–Cu_2_O modified electrode was obtained using CA technique with the stepwise addition of glucose at every 50 s (Fig. 8a). A quick change in current (less than 5 s) was observed with the addition of glucose to the electrolyte. A magnified image at the lowest concentration of glucose and the respective changes in current is provided in the inset of Fig. 8a. Even for the lower concentration (10 µM), there is an enhancement in the current. Later, a linear plot of current vs. concentration of glucose is obtained as given in Fig. 8b. Similarly, M–Cu_2_O (Fig. 8b) and rGO–Cu_2_O (Fig. S9a) composites were studied and the corresponding current response with respect to the change in concentration is given in Figs. 8c, S9b respectively. MG–Cu_2_O composite shows a wider linear range as compared to rGO–Cu_2_O composites whereas it is similar as M–Cu_2_O composite. However, a tenfold increment of current is observed by the replacement of M–Cu_2_O with MG–Cu_2_O during the sensing of glucose molecules. It might be due to the lowest d-spacing and highest defect density of the prepared structure as well as the introduction of rGO, which avoids the restacking of MXene layers and enhance the active sites as compared to M–Cu_2_O. Regression equations for M–Cu_2_O and rGO–Cu_2_O are ΔI(µA) = 0.785 [Glucose] mM + 0.3623 with R2 = 0.99, ΔI (µA) = 1.174 [Glucose] mM + 0.5 with R2 = 0.97, respectively.

Moreover, CA response of the MG–Cu_2_O in glucose environment is divided into two sections of linearity that is 0.01 to 10 mM and 11 to 30 mM with the slope value of 8.992 and 6.323, respectively. The corresponding regression equations in these two sections are ΔI (µA) = 8.992 [Glucose] mM + 1.73 with R^2^ = 0.99 and ΔI (µA) = 6.323 [Glucose] mM + 28.3 with R^2^ = 0.99. From these equations, a sensitivity of M–Cu_2_O, rGO–Cu_2_O and MG–Cu_2_O is calculated and it given in Fig. 8d. A table of comparison of our material with the reported ones is given in Table 1.

**Table 1:** A comparison of glucose sensing performance of MG–Cu_2_O with others glucose sensors.

Electrode	Sensing method	Linear range (mM)	Detection limit (µM)	Sensitivity (µAmM^−1^ cm^−2^)	Ref
Cu_2_O/Au/GO	NE	Upto 16.65	0.83	2886	^34^
CuO/CG-GCE	NE	0.1–3.17	0.01	1295	^66^
Cu NP on laser induced graphene	NE	0.001–6	0.39	495	^67^
Ni/rGO/PU	NE	0.01–2	1.28	4876	^68^
Cu-Co/rGO/PGE	NE	0.001–4	0.15	240	^69^
S doped rGO/CuS	NE	20.1	0.032	429	^65^
Pt-CuO-Pt	NE	2.2–10	1.42	2921	^18^
Cu/rGO	NE	0.1–12.5	65	172	^70^
GO_x_/Au/MX/Nafion	E	0.1–18	5.9	4.2	^43^
NiO/Graphene	NE	0.005–4.2	0.1	666.7	^33^
MXene/NiCo-LDH	NE	0.002–4.096	0.53	64.75	^71^
MXene-Cu_2_O	NE	0.01–30	2.83	11.064	^44^
Cu_2_O/rGO/MXene (MG-Cu_2_O)	NE	0.01–30	2.1	125.6	This work

*GCE glassy carbon electrode, *CG* carboxylate graphene, *PU* polyurethane, *PCE* pencil graphite electrode, *LDH* layered double hydroxide, *NE* non enzymatic, *E* enzymatic.

From Fig. 8d and Table 1, it could be understood that the prepared ternary composite (MG–Cu_2_O) is having the enhanced sensitivity with a wide linear range of glucose sensing as compared to other sensing elements (rGO–Cu_2_O and M–Cu_2_O). In these composites, a large surface area, high conductivity (6500 S cm^−1^) and hydrophilicity nature of MXene sheet will increase the CuOOH formation in the electrolyte. It might generate the enediol and lower the energy barrier of electrooxidation reaction with the addition of glucose to the electrolyte. This will increase the number of electrons leading to a wide linear range as per the Yang et al.^71^. But the sensitivity of the sensor was low as per the requirement for commercialization. Restacking of MXene sheet may reduce the rate of electron transfer between the electrode and electrolyte. To prevent this, an incorporation of rGO sheet (rGO will improve the rate of electron transfer process during the glucose sensor^33^) to MXene was carried out. It will maintain the larger surface area of sheets as well as the ample amount of functional groups on the surface and edges of sheet may hinder the nanoparticle agglomeration leading to the formation of spherical shape of Cu_2_O in the MG–Cu_2_O composite, which further enhances the CuOOH formation and increases the rate of electron transfer between electrode and electrolyte during the oxidation of glucose. In the prepared ternary composites, MXene improves the linear range by a greater number of CuOOH formation and rGO will improve the rate of electron transfer between the electrode and electrolyte. The combined role of MXene and rGO has thus promoted the sensitivity of Cu_2_O composite with wide linear range.

### Interference, stability and reproducibility of MG–Cu_2_O modified electrode

**Figure 9:**
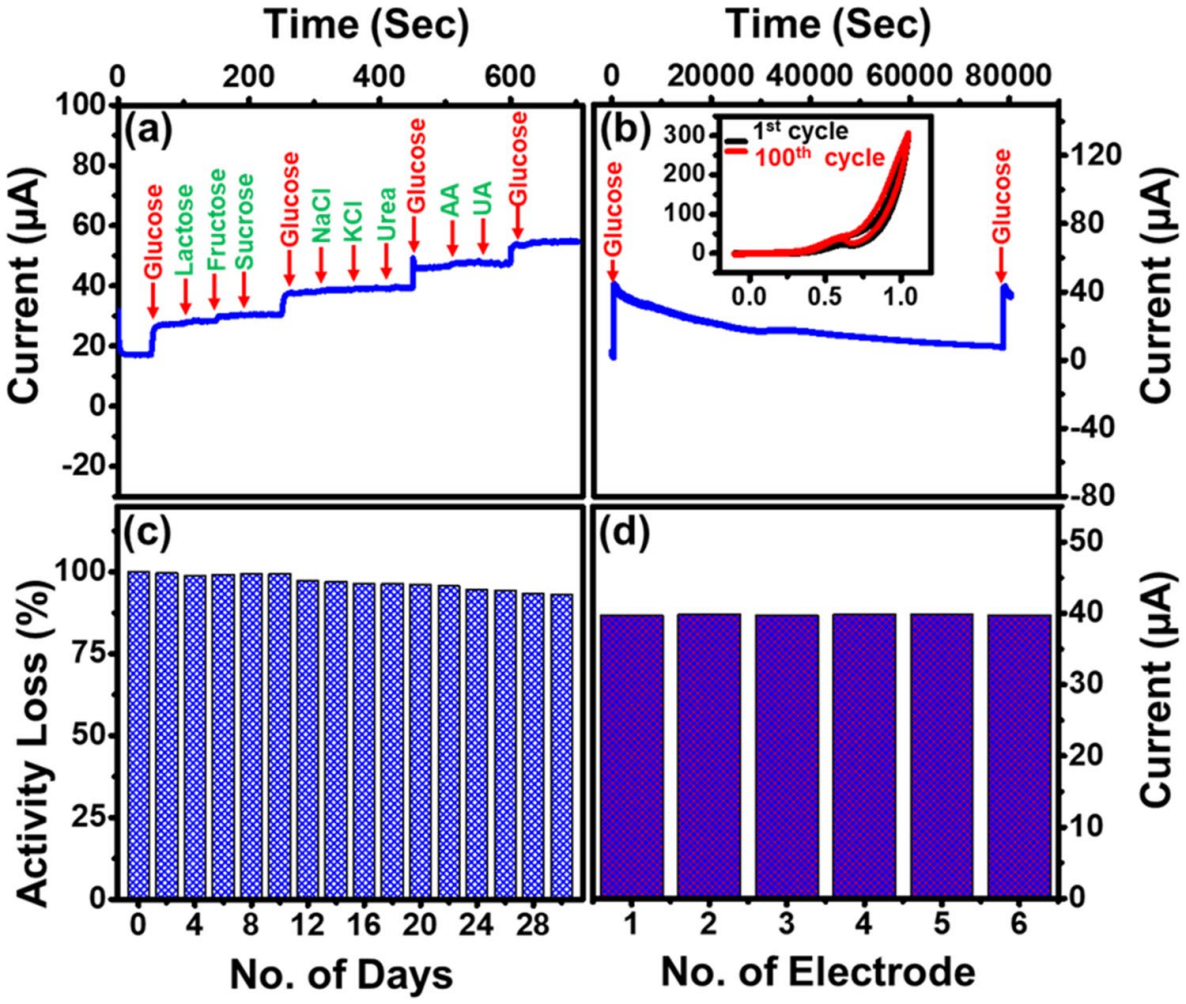
(**a**) Sensing analysis of respective elements in human serum for MG–Cu_2_O (0.5 mM glucose along with 0.05 mM of other active species) (**b**) stability study of electrode in electrolyte using CV (inset) and CA (**c**) storage stability of modified electrode with 0.1 mM of glucose (**d**) Sensing response of six different modified electrodes with MG–Cu_2_O composite.

To understand the interference effect of MG–Cu_2_O composite, CA study was carried out with other interference species including ascorbic acid, uric acid, lactose, fructose, sucrose, NaCl, KCl and urea along with glucose molecules**.** The concentration of glucose and other interference species are used for this analysis as similar to the physiological level of glucose in human blood^64^. From Fig. 9a, a massive and abrupt change in current was observed for the glucose molecule as compared to other species. It indicates that the prepared composite is having a good anti-interference effect. Moreover, stability and durability of MG–Cu_2_O modified electrode was carried out both by chronoamperometry and cyclic voltammetry techniques.

A CV study was done in the presence of 10 mM glucose in the electrolyte for 100 cycles. It can be observed from the inset of Fig. 9b, that even after 100 cycles, the peak current was maintained as similar to the initial value. Along with this, CA test was carried out in the presence of glucose (1 mM) over time duration of 20 h. It is observed from Fig. 9b that initially a huge change in current was observed with the addition of glucose and gradually decreased over time due to the oxidation of glucose in the electrolyte. To confirm this phenomenon, an equal amount of glucose was subsequently added to the electrolyte and the change of current is detected as similar to the initial change, which indicates that the MG–Cu_2_O modified electrode has a good operational stability. Furthermore, durability study was carried out by storing the modified electrode in ambient atmosphere for 30 days. To examine this, a change in current in 0.1 mM glucose is analyzed for two days once and then stored at ambient atmosphere. This process is repeated until 30 days and then the activity loss of the device is evaluated and given in Fig. 9c. After 30 days, only 10% of loss in the current from its initial value was observed, which signifies the good durability of the prepared electrode. Moreover, the reproducibility of MG–Cu_2_O modified electrode was examined by performing CA test with six different electrodes and demonstrated in Fig. 9d. A similar change in current is observed in all the cases, which confirmed the reproducible nature of the sensing material.

### Real time performance of MG–Cu_2_O modified electrode

To understand the real time application of the prepared sensing electrode, the electrochemical test of the prepared sensing element in the presence of three different serums was investigated. It was observed that the present sensing electrode shows 7.01 mM concentration of glucose with the addition of serum 1, whereas it was found 7.15 mM concentration of glucose using a commercial glucometer. A detailed relative standard deviation value between the proposed and commercial glucometer is tabulated in Table 2. Lower value of deviation suggests that the prepared electrode is well suitable for real time application with good reliability.

**Table 2:** Real time analysis of MG–Cu_2_O based electrode (Human serum).

Serum	Concentration of glucose as sensed by		Relative standard deviations (RSD %)
	Proposed sensor (mM)	Commercial glucometer (mM)	
1	7.01	7.15	1.4
2	5.71	5.89	2.19
3	6.1	6.31	2.39

## Conclusion

In this report, the role of graphene oxide in MXene–rGO composites is optimized and investigated as a sensing probe for glucose sensing application. The optimized MG composite is later utilized to prepared the ternary composite of rGO supported MXene based Cu_2_O composite (MG–Cu_2_O) by coprecipitation method. Structural analysis confirmed that the prepared composite is having high defect density with lower crystalline size as compared to M–Cu_2_O composite. Such composites might lead to enhance the conductivity and amplifies the signal towards glucose oxidation with MG–Cu_2_O as compared to M–Cu_2_O. Therefore, the prepared composite shows a sensitivity of 125.6 µAmMcm^−1^ with a broad linear range (0.01 to 30 mM) and further it shows a good selectivity, stability and reproducibility. Results show that the prepared MG–Cu_2_O composite is preferable for analyzing the glucose level in human serum.

## Supplementary Information

Supplementary Information.

## Data Availability

All data generated or analysed during this study are included in this published article.
