# Supplementary material for: Reduced graphene oxide supported MXene based metal oxide ternary composite electrodes for non-enzymatic glucose sensor applications
Source: Sci Rep. 2022 Nov 29;12:20583. doi: 10.1038/s41598-022-24700-w (PMC9708649; doi:10.1038/s41598-022-24700-w)
Supplement: Supplementary file 1 — Supplementary Information. [file 41598_2022_24700_MOESM1_ESM.docx]

**Supplementary Information**


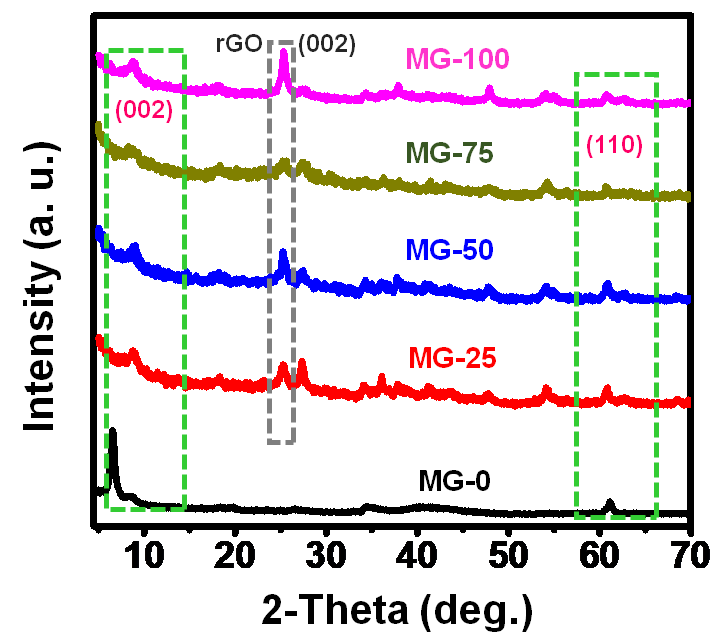


**Figure S1.** X-Ray diffraction of MXene with different weight ratio of graphene oxide.


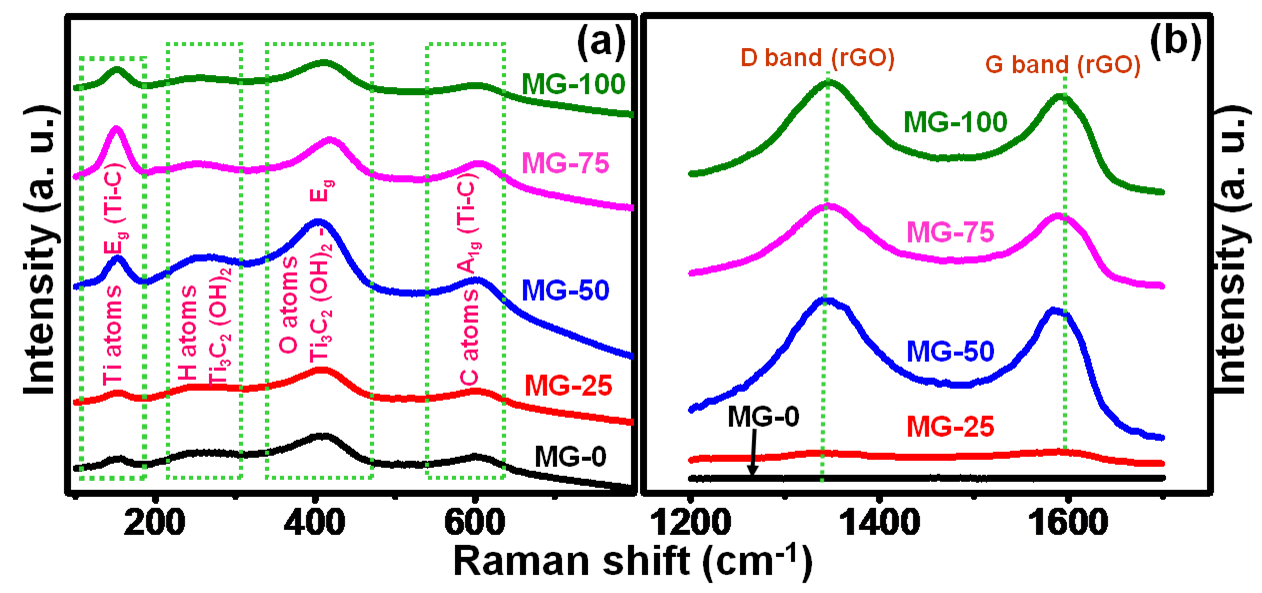
**Figure S2.** (a) Raman spectra of MXene at different weight ratio of graphene oxide (b) D and G band of MXene with different weight ratio of graphene oxide.


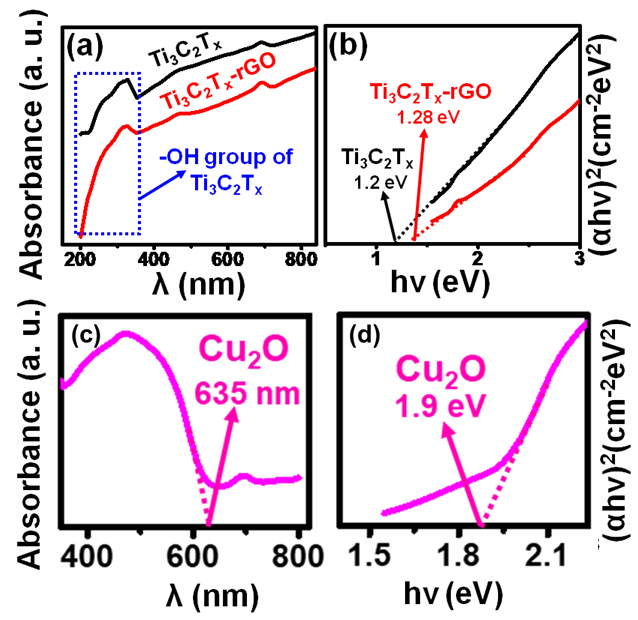


**Figure S3.** UV absorption spectra of (a) MXene and MXene with reduced graphene oxide (b) Tauc plot of MXene and MXene with reduced graphene oxide.


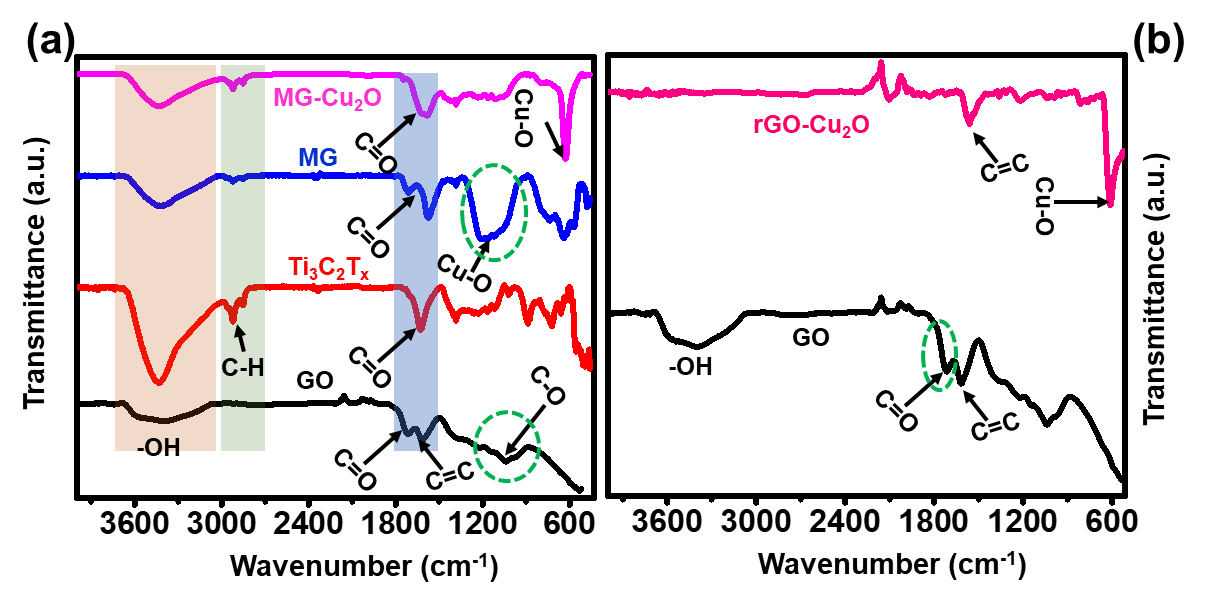


**Figure S4.** FTIR of (a) bare and MXene based composite (b) GO and rGO-Cu_2_O composites.


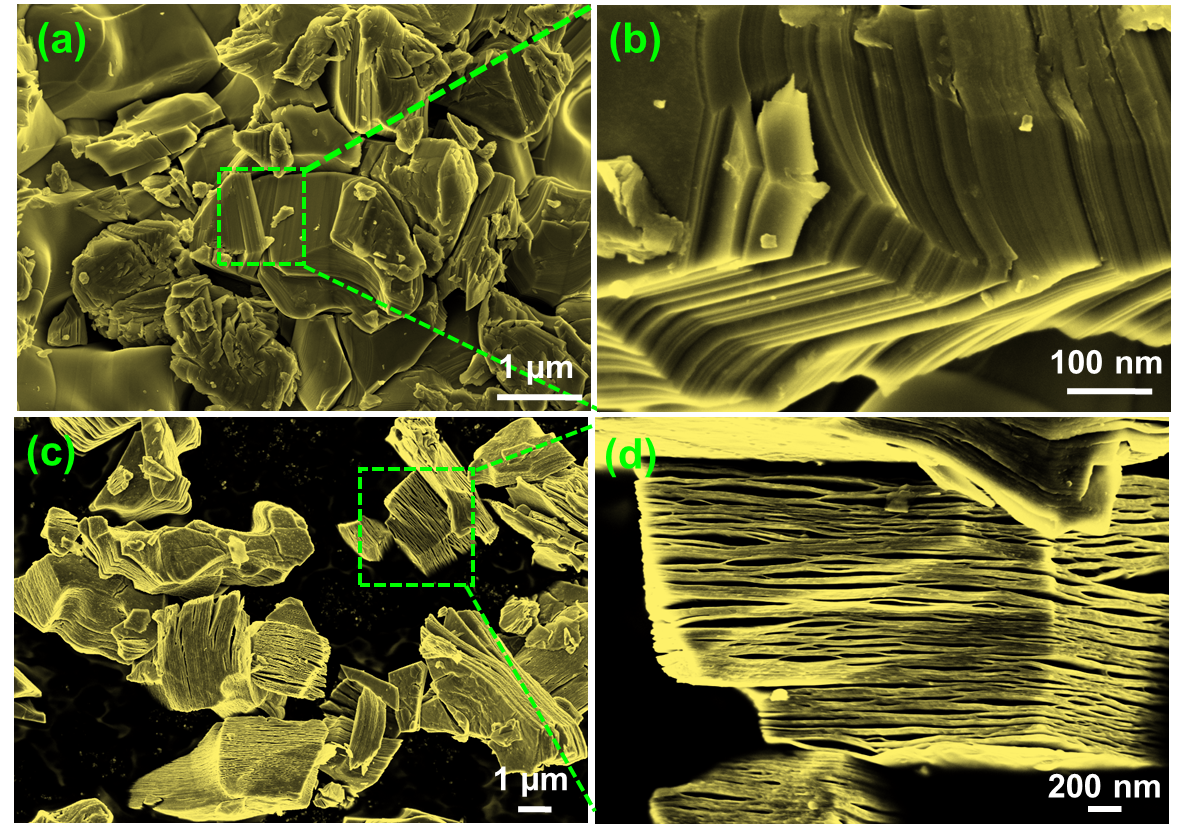


**Figure S5.**  FE-SEM images at different magnifications (a, b) MAX phase, and (c, d) MXene.


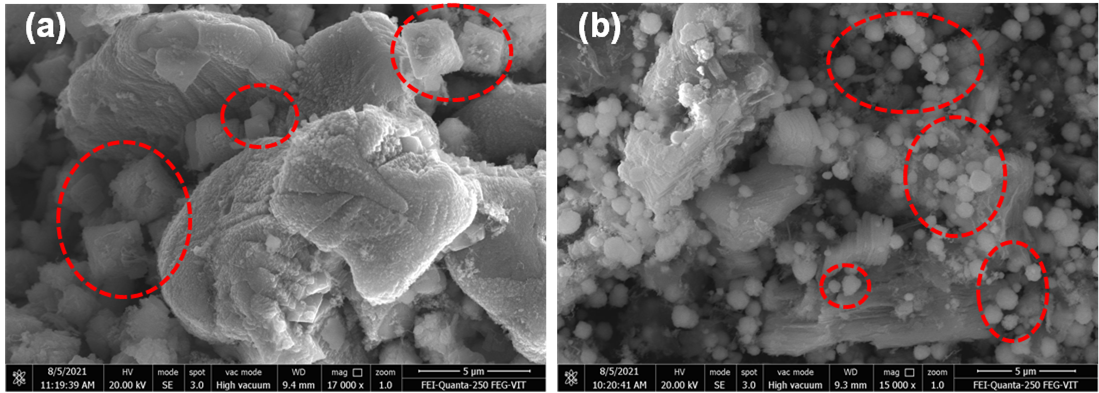


**Figure S6.** FE-SEM image of (a) M-Cu_2_O composite and (b) MG-Cu_2_O composite.


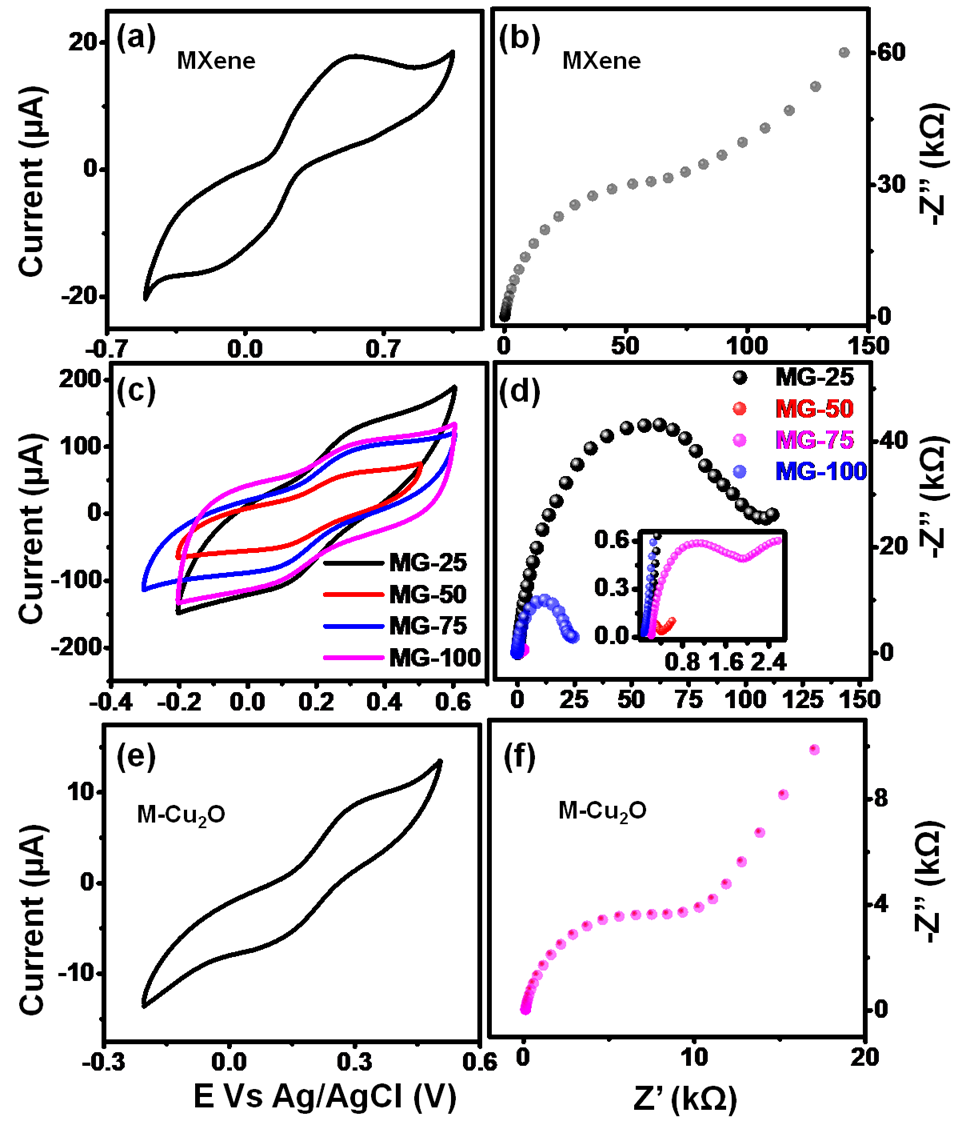


**Figure S7.** CV profile of (a) MXene (c) MG composite with different weight ratio (e) M - Cu_2_O, EIS of (b) MXene (d) MG composite with different weight ratio and (f) M - Cu_2_O. (All the studies are done with 0.1 M of KCl +5 mM of Fe (CN)^3-/4-^ solution)


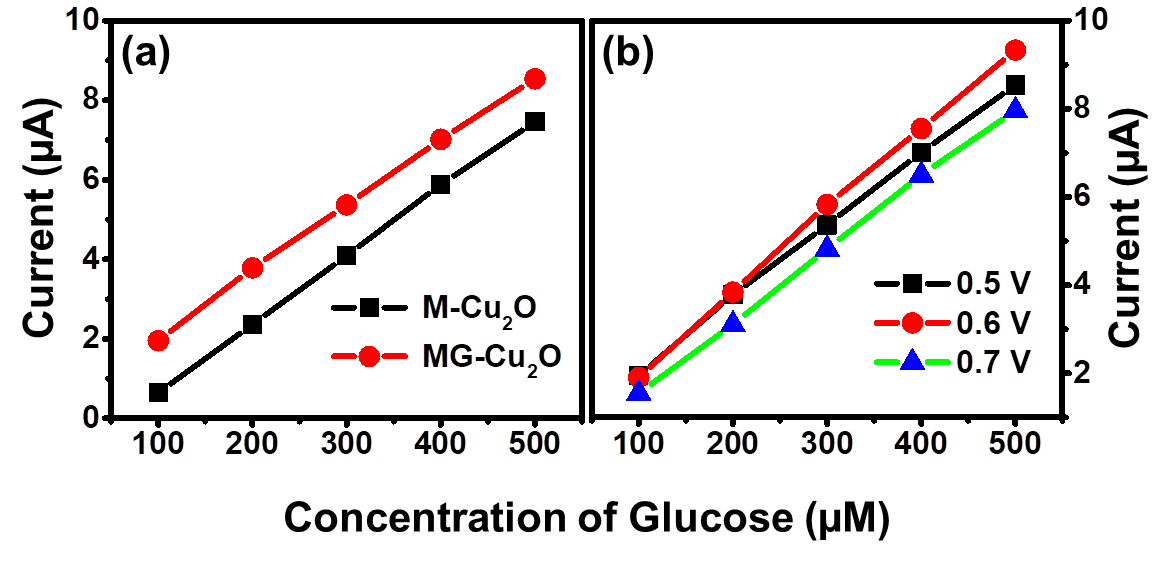


**Figure S8.** A linear plot of current Vs glucose concentration from the CA study (a) for various prepared materials (M-Cu_2_O and MG-Cu_2_O) (b) at the different bias voltages for MG-Cu_2_O composite in 0.1 M NaOH.


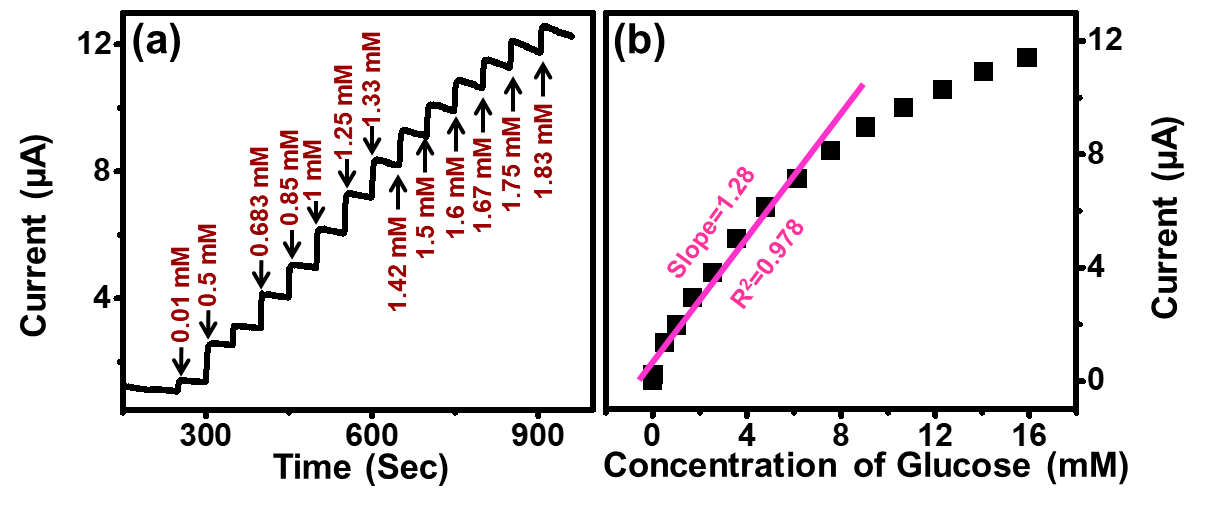


**Figure S8.** (a) CA study of rGO-Cu_2_O composite with different addition of glucose in 0.1 M NaOH (b) Linear Plot of rGO-Cu_2_O.

| **S.No.** | **Sensing element** | **Linear Range (mM)** | **Detection limit (uM)** | **Sensitivity**  **(mA mM^-1^cm^-2)^** | **Ref. No.** |
| --- | --- | --- | --- | --- | --- |
| 1 | 3D CuO micro nanofibers (Morphology variation) | Upto 1.43 | 7.07 | 0.023 | 1 |
| 2 | Nano copper oxide micro hollow sphere (Size and Shape variation) | Two linear range   - 1. to 3,   3 to 11.5 | - | 1. 0.025  2. 0.0136 | 2 |
| 3 | Cu_2_O shuriken-like nanostructures (Morphology variation) | 0.01 to 11 mM | - | 933 | 3 |
| 4. | 26-Facet Cu_2_O or rhombicuboctahedra Cu_2_O (Face Tunning) | 0.05-4 | 0.41 | 1.53 | 4 |
| 5. | Cu_2_O/rGO (Composite) | 0.01-9 | 0.44 | 0.814 | 5 |
| 6. | Octohedron Cu_2_O | 0.1 to 5 | 5.11 | 0.294 | 6 |
| 7. | Nano- and Microstructured Copper/Copper Oxide Composites on Laser-Induced Carbon | 0.001 to 3.3 | 1.75 | 0.32 | 7 |
| 8. | Electrodeposited Cu_2_O/ITO electrode (Deposition variation) | 0.04 to 0.4 | 4 | 2.2 | 8 |
| 9. | Cuprous oxide (Cu_2_O) nanoparticles at a loading of 33.5 wt.% were impregnated in amine-functionalized mesoporous silica (NH2-SBA-15) particles | 0.2 to 15 | - | 0.438 | 9 |
| 10 | 3D porous Cu@Cu_2_O aerogels (Morphology variation) | 0.001 to 5.2 mM, 5.2 to 17.1 mM | 0.6 | 195 | 10 |

Table S1. A table of comparison of the sensing performance of Cu_2_O based metal oxides with several form of variation such as morphology, face, size, deposition process and nanocomposites.

**References**

1. Liu, M. *et al.* A non-enzymatic glucose sensor based on electrospun 3-D copper oxide micro-nanofiber network films using carboxylic-functionalized poly (arylene eher ketone)s as templates. *RSC Advances***9**, 6613-6619 (2019).
2. Haghparas, Z. *et al.* Fabrication of non-enzymatic electrochemical glucose sensor based on nano-copper oxide micro hollow spheres. *Biotechnology and Bioprocess Engineering***25**, 528-535 (2020).
3. Khan, R. *et al.* Glucose-assisted synthesis of Cu_2_O shriken-like nanostructures and their application as nonenzymatic glucose biosensor, *Sensors and Actuators B: Chemical***203**, 471-476 (2014).
4. Ren, H. *et al.* An Mn^2+^-mediated construction of rhombicuboctahedral Cu_2_O nanocrystals enclosed by jagged surfaces for enhanced enzyme-free glucose sensing, *CrystEngComm***22**, 2042-2048 (2020).
5. Gijare, M *et al.* Reduced Graphene oxide based electrochemical nonenzymatic human serum glucose sensor, *ES Materials and Manufacturing***14**, 110-119 (2021).
6. Rakshit, S *et al.* Non-enzymatic electrochemical glucose sensing by Cu_2_O octahedrons: elucidating the protein adsorption signature, *New Journal of Chemistry***45**, 628-637 (2021).
7. Mamleyev R- E. *et al.* Nano- and microstructured copper/copper oxide composites on laser-induced carbon for enzyme-free glucose sensors, Applied Nano Materials**4**, 13747-13760 (2021).
8. Laidoudi S. *et al.* Non-enzymatic glucose detection based on cuprous oxide thin film synthesized via electrochemical deposition, Applied Physics A**160**, 1-10 (2020).
9. Khan, Y- A. *et al.* Highly sensitive non-enzymatic glucose sensor with copper oxide nanoparticle impregnated mesoporous silica, *Journal of Porous Materials* **28**, 1097-1104 (2021).
10. Gao, Y. *et al.* Three-dimensional porous Cu@Cu_2_O aerogels for direct voltametric sensing of glucose, *Microchimica Acta***186**, 1-9 (2019).
